# Early 5‐HT_6_ receptor blockade prevents symptom onset in a model of adolescent cannabis abuse

**DOI:** 10.15252/emmm.202216481

**Published:** 2022-09-07

**Authors:** Coralie Berthoux, Al Mahdy Hamieh, Angelina Rogliardo, Emilie L Doucet, Camille Coudert, Fabrice Ango, Katarzyna Grychowska, Séverine Chaumont‐Dubel, Pawel Zajdel, Rafael Maldonado, Joël Bockaert, Philippe Marin, Carine Bécamel

## Abstract

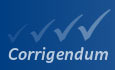

**Correction to:** *EMBO Mol Med* (2020) 12: e10605. DOI: 10.15252/emmm.201910605 ¦ Published online 24 April 2020

The authors wish to correct electrophysiological and behavioral data shown in Figs 1E, and 5E and I, and the corresponding Materials and Methods.

**Figure 1E**: The authors correct the labeling of the *Y*‐axis in the bottom panel of Fig 1E.

**Figure:**
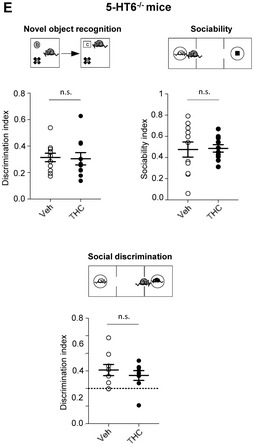
Figure 1E. Original.

**Figure:**
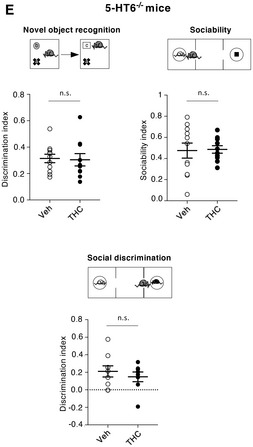
Figure 1E. Corrected.

**Figure 5E**: A scale bar has been added to Fig 5E.

**Figure:**
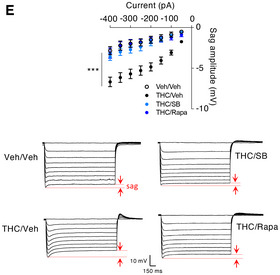
Figure 5E.

**Figure 5I**: Some data points from the Veh and THC plots in 5D were incorrectly copied into 5I, which slightly affected the mean without affecting the statistics. We are herewith retracting 5I and replacing it with the corrected figure. The authors note that the interpretation of the data remains the same.

**Figure:**
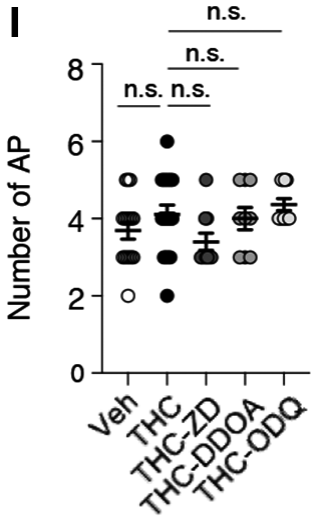
Figure 5I. Original.

**Figure:**
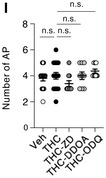
Figure 5I. Corrected.

In addition, two sentences in the Electrophysiological recordings section of Materials and Methods are updated:

The sentence,

“Action potentials (APs) were evoked by a ramp current injection with 10‐pA increments for 2 ms, and only the first AP was used to determine the AP threshold and the minimal current necessary to induce firing (rheobase).”

Is updated to

“Action potentials (APs) were evoked by a ramp current injection with 10‐pA increments for 2 ms to determine the AP threshold and the minimal current necessary to induce firing (rheobase).”

The sentence,

“Hyperpolarization‐activated cation current (Ih) was measured as the voltage sag to a 500 ms hyperpolarizing current injection (50‐pA increment from −400 pA to 0).”

Is updated to

“Hyperpolarization‐activated cation current (Ih) was measured as the voltage sag to a 2,000 ms hyperpolarizing current injection (50‐pA increment from −400 pA to 0).”

The authors acknowledge the constructive input from Sodikdjon Kodirov and apologize for these errors and any confusion they may have caused.

