# Supplementary material for: Early 5‐HT 6 receptor blockade prevents symptom onset in a model of adolescent cannabis abuse
Source: EMBO Mol Med. 2020 Apr 24;12(5):e10605. doi: 10.15252/emmm.201910605 (PMC7207164; doi:10.15252/emmm.201910605)
Supplement: Supplementary file 1 — Appendix [file EMMM-12-e10605-s001.pdf]

Table of contents:

5 Appendix Figures (Figure S1 to S5)

Legends of the Appendix Figures

Appendix Table S1

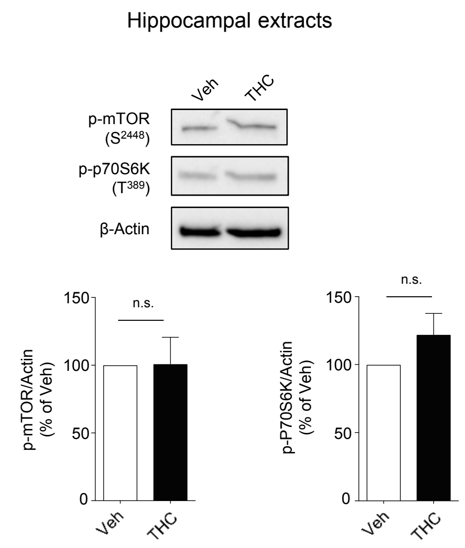

Appendix Figure S1

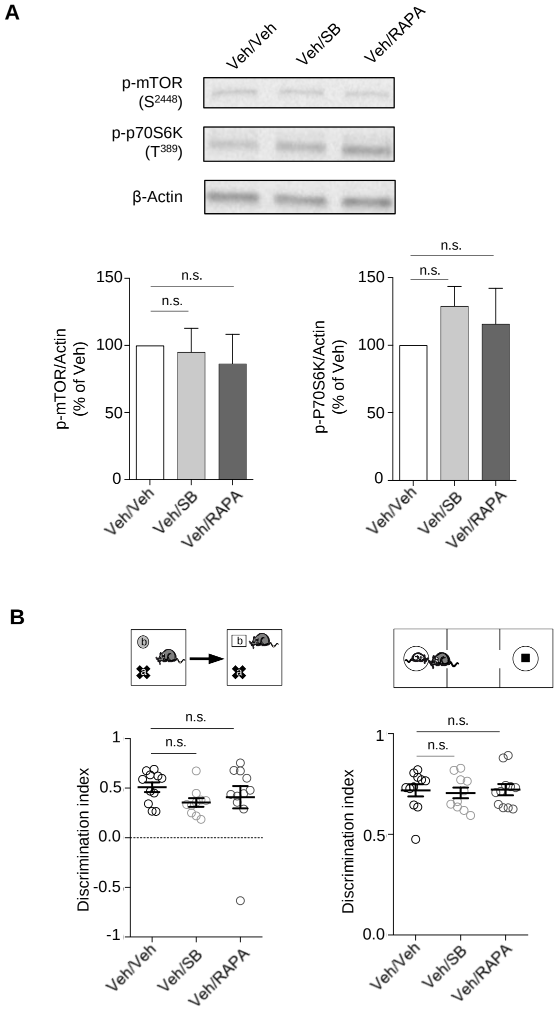

Appendix Figure S2

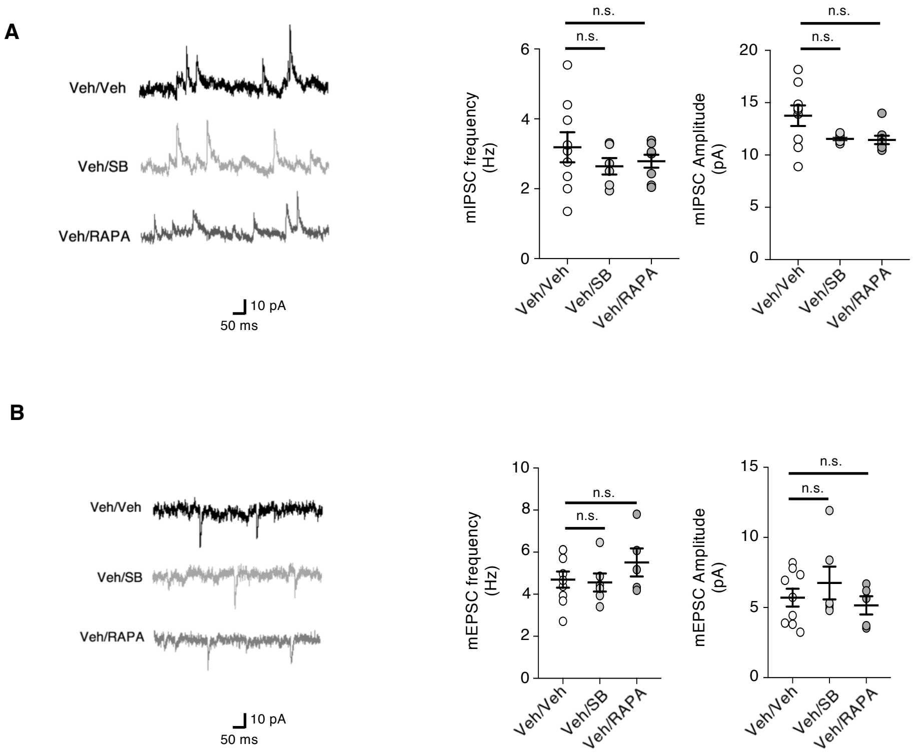

Appendix Figure S3

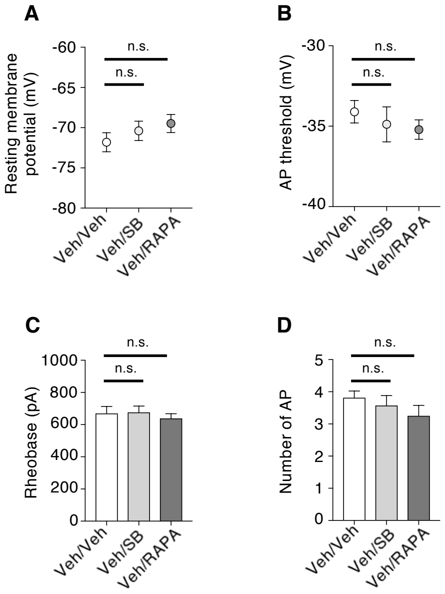

Appendix Figure S4

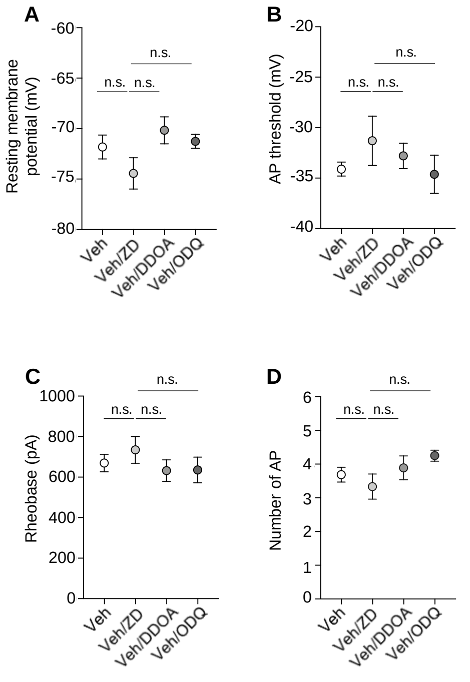

Appendix Figure S5

Supplementary Legends

Appendix Figure S1. Chronic consumption of THC during adolescence does not induce mTOR activation in hippocampus of adult mice

Mice were injected daily with THC (5 mg/kg) or vehicle (Veh) during adolescence, from PND 30 to 45. Top: representative Western blots assessing mTOR phosphorylation at S2448 and p70S6K phosphorylation at T389 as indexes of mTOR activity in the hippocampus of adult mice are illustrated. Bottom: data represent the ratios of immunoreactive signals of the anti phospho-mTOR (S2448) or anti phospho-P70S6K (T389) antibodies to the immunoreactive signal of the anti-🚲-actin antibody and are expressed in % of values in vehicle-injected mice. They are the means ± S.E.M. of results obtained in four mice per group. n.s. $p > 0.05$, unpaired Student's t test.

Appendix Figure S2. Administration of SB258585 or Rapamycin during adolescence does not affect prefrontal mTOR activity and cognitive performance in adult mice

Mice were injected daily with either vehicle (Veh) or SB258585 (SB, 2.5 mg/kg) or rapamycin (Rapa, 1.5 mg/kg) from PND 30 to 45. Biochemical and behavioral experiments were performed from PND 60. A. Top: representative Western blots assessing mTOR phosphorylation at S2448 and p70S6K phosphorylation at T389 as indexes of mTOR activity in PFC of adult mice are illustrated. Bottom: data represent the ratios of immunoreactive signals of the anti phospho-mTOR (S2448) or anti phospho-p70S6K (T389) antibodies to the immunoreactive signal of the anti-🚲-actin antibody and are expressed in % of values in vehicle-injected mice. They are the means ± S.E.M. of results obtained in four mice per group. n.s. $p > 0.05$, one-way ANOVA followed by Newman-Keuls test. B. Top: schemas illustrating the

behavioral tasks examined. Bottom: the plots represent the discrimination index measured in each condition. n.s. $p > 0.05$, one-way ANOVA followed by Bonferroni test. Discrimination index for the novel object discrimination: 0.36 ± 0.04 and 0.41 ± 0.11 for Vehicle+SB258585 (N=10) and Vehicle+Rapa (N=11), respectively, $p > 0.05$ vs. Vehicle-injected mice. Sociability index: 0.70 ± 0.02 and 0.72 ± 0.02 for Vehicle+SB258585 and Vehicle+Rapa conditions, respectively, $p > 0.05$ vs. Vehicle-injected mice. Social discrimination index: 0.23 ± 0.09 and 0.20 ± 0.11 for Vehicle+SB258585 (N=6) and Vehicle+Rapa (N=5) conditions, respectively, $p > 0.05$ vs. Vehicle-injected mice.

Appendix Figure S3. Administration of SB258585 or Rapamycin during adolescence has no effect on inhibitory and excitatory synaptic transmissions in the PFC of adult mice.

Mice were injected daily with either vehicle (Veh) or SB258585 (SB, 2.5 mg/kg) or rapamycin (Rapa, 1.5 mg/kg) from PND 30 to 45. Electrophysiological recordings were performed from PND 60. **A.** Left: representative traces of GABA mIPSCs recorded in layer V pyramidal neurons are illustrated. Right: the histograms represent means ± S.E.M. of GABA mIPSC frequency and amplitude measured during the last minute of recording. n=6 from N=3 for Veh.+SB and n= 8 from N=3 for THC+Rapa. **B.** Left: representative traces of AMPA mEPSCs are illustrated. Right: the histograms represent means ± S.E.M. of AMPA mEPSC frequency and amplitude measured during the last minute of recording. n=6 from N=4 for Veh.+SB and n= 5 from N=4 for THC+Rapa. n.s. $p > 0.05$, one-way ANOVA followed by Tukey test.

Appendix Figure S4. Administration of SB258585 or Rapamycin during adolescence does not alter the intrinsic properties of layer V pyramidal neurons. Mice were injected daily with either vehicle (Veh) + vehicle (Veh/Veh) or SB258585 (Veh/SB, 2.5 mg/kg) or rapamycin

(Veh/Rapa, 1.5 mg/kg) from PND 30 to 45. Electrophysiological recordings were performed from PND 60. A-D. The histograms represent means ± S.E.M. of RMPs, AP thresholds, rheobases and firing rates (measured as described in the legend to Figure 4), respectively. n.s. $p > 0.05$, one-way ANOVA followed by Tukey test. n=15 from N=5 for Veh/ SB and n= 16 from N=5 for Veh/Rapa. RMP: -70.4 ± 1.2 mV and -69.5 ± 1.1 mV for Veh/SB and THC/Rapa conditions, respectively; AP threshold: -34.9 ± 1.1 mV and -35.2 ± 0.6 mV for Veh/SB and THC/Rapa conditions, respectively; Rheobase: 675 ± 39 pA and 639 ± 29 pA for Veh/SB and THC/Rapa conditions, respectively.

Appendix Figure S5. Blocking HCN1 channel, adenylyl cyclase or guanylyl cyclase activity does not modify intrinsic properties of layer V pyramidal neurons from vehicle-injected mice.

A-D. The resting membrane potential (RMP, A), AP threshold (B), rheobase (C) and firing rate (D) were determined in acute PFC slices from vehicle-injected mice after 5-min application of the HCN1 channel blocker ZD7288 (ZD, 10 µM, n=9 from N=3) in the recording chamber or DDOA (15 µM, n=9 from N=4) or ODQ (10 µM, n=9 from N=4) into the recording pipette, as described in the legend to Figure 5. The histograms represent the means ± S.E.M of RMPs, AP thresholds, rheobases and firing rates, respectively. n.s. $p > 0.05$, one-way ANOVA followed by Tukey test. RMP: -71.8 ± 1.2 mV; -74.4 ± 1.6 mV; -70.2 ± 1.3 mV and -71.3 ± 0.7 mV for Veh, Veh/ZD, Veh/DDOA and Veh/ODQ, respectively; AP threshold: -34.1 ± 0.7 mV, -31.3 ± 2.4 mV; -32.8 ± 1.3 mV and -34.6 ± 1.9 mV for Veh, Veh/ZD, Veh/DDOA and Veh/ODQ, respectively; Rheobase: 670 ± 43 pA; 734 ± 66 pA; 632 ± 53 pA and 636 ± 63 pA for Veh/ZD, Veh/DDOA and Veh/ODQ, respectively.

Appendix Table S1: Detailed statistics for figures

**Figure #**	**Analysis**	**Statistics**
Fig. 1B P-mTOR	1-way ANOVA	$F(3,16)$= 5.220; P=0.0105
Fig. 1B P-p70	1-way ANOVA	$F(3,16)$= 4.585; P=0.0168
Fig. 1C (NOR)	1-way ANOVA	$F(3, 42)$= 5.626; P=0.0025
Fig. 1C (sociability)	1-way ANOVA	$F(3, 41)$= 7.95; P=0.0003
Fig. 1C (social discrimination)	1-way ANOVA	$F(3, 28)$= 5.031; P=0.0065
Fig. 1D P-mTOR	2-tailed unpaired *t* test	$P=0.1976$
Fig. 1D P-p70	2-tailed unpaired *t* test	$P=0.7646$
Fig. 1E (NOR)	2-tailed unpaired *t* test	$P=0.8629$
Fig. 1E (sociability)	2-tailed unpaired *t* test	$P=0.8960$
Fig. 1E (social discrimination)	2-tailed unpaired *t* test	P=0.4792
Fig. 3B P-mTOR	1-way ANOVA	$F(3, 20)$= 7.281; P=0.0017
Fig. 3B P-p70	1-way ANOVA	$F(3, 20)$= 3.917; P=0.0238
Fig. 3C	1-way ANOVA	$F(3, 69)$= 7.159; P=0.0003
Fig. 4B Frequency	1-way ANOVA	$F(3, 32)$= 13.39; P<0.0001
Fig. 4B Amplitude	1-way ANOVA	$F(3, 32)$= 10.08; P=0.7010
Fig. 4C Frequency	1-way ANOVA	$F(3, 28)$= 5.733; P=0.0034
Fig. 4C Amplitude	1-way ANOVA	$F(3, 28)$= 0.9516; P= 0.4292
Fig. 5A	1-way ANOVA	$F(3, 67)$= 19.7; P< 0.0001
Fig. 5B	1-way ANOVA	$F(3, 67)$= 8.225; P< 0.0001
Fig. 5C	1-way ANOVA	$F(3, 67)$= 11.34; P< 0.0001
Fig. 5D	1-way ANOVA	$F(3, 64)$= 1.647; P= 0.1873
Fig. 5E	1-way ANOVA	$F(4, 63)$= 18.78; P< 0.0001
Fig. 5F	1-way ANOVA	$F(4, 63)$= 5.366; P= 0.0009
Fig. 5G	1-way ANOVA	$F(4, 64)$= 14.93; P< 0.0001
Fig. 5H	1-way ANOVA	$F(4, 59)$= 2.256; P=0.0738
Fig. 6B	1-wayANOVA	$F(2, 6)$= 8.357; P= 0.0184
Fig. S1 P-mTOR	2-tailed unpaired *t* test	$P=0.9624$
Fig. S1 P-p70	2-tailed unpaired *t* test	$P=0.2103$
Fig. EV1A P-mTOR	2-tailed unpaired *t* test	$P=0.0704$
Fig. EV1A P-p70	2-tailed unpaired *t* test	$P=0.5577$
Fig. EV1B P-mTOR	2-tailed unpaired *t* test	$P=0.6737$
Fig. EV1B P-p70	2-tailed unpaired *t* test	$P=0.6252$
Fig. S2A P-mTOR	1-way ANOVA	$F(2,12)$= 0.1786; P=0.8386
Fig. S2A P-p70	1-way ANOVA	$F(2,12)$= 0.6959; P=0.5177
Fig. S2B (NOR)	1-way ANOVA	$F(2, 29)$= 1.020; P=0.3732
Fig. S2B (sociability)	1-way ANOVA	$F(2, 29)$= 0.08611; P=0.9177
Fig. S2B (social discrimination)	1-way ANOVA	$F(2, 16)$= 0.6117; P=0.6117
Fig. EV2A open entries	2-tailed unpaired *t* test	$P=0.1824$
Fig. EV2A center	2-tailed unpaired *t* test	$P=0.5719$
Fig. EV2B	2-tailed unpaired *t* test	$P=0.1462$

Fig. EV3B P-mTOR	2-tailed unpaired *t* test	*P=0.8362*
Fig. EV3B P-p70	2-tailed unpaired *t* test	*P=0.6313*
Fig. EV3C (NOR)	1-way ANOVA	$F(3, 33)$= 8.343; P=0.0003
Fig. S3A Frequency	1-way ANOVA	$F(2, 17)$= 0.9834; P=0.3943
Fig. S3A Amplitude	1-way ANOVA	$F(2, 17)$= 0.8034; P=0.4641
Fig. S3B Frequency	1-way ANOVA	$F(2, 20)$= 0.7390; P=0.4902
Fig. S3B Amplitude	1-way ANOVA	$F(2, 20)$= 0.51; P=0.6047
Fig. S4A	1-way ANOVA	$F(2, 46)$= 1.044; P=0.3603
Fig. S4B	1-way ANOVA	$F(2, 46)$= 0.5266; P= 0.5941
Fig. S4C	1-way ANOVA	$F(2, 46)$= 0.2557; P=0.7755
Fig. S4D	1-way ANOVA	$F(2, 39)$= 1.005; P=0.3755
Fig. EV4A	2-tailed unpaired *t* test	*P=0.9687*
Fig. EV4B	2-tailed unpaired *t* test	*P=0.8964*
Fig. EV4C	2-tailed unpaired *t* test	*P=0.3917*
Fig. EV4D	2-tailed unpaired *t* test	*P=0.7943*
Fig. S5A	1-way ANOVA	$F(3, 41)$= 1.582; P=0.2084
Fig. S5B	1-way ANOVA	$F(3, 41)$= 0.9422; P= 0.4291
Fig. S5C	1-way ANOVA	$F(3, 41)$= 0.5999; P=0.6188
Fig. S5D	1-way ANOVA	$F(3, 38)$= 1.509; P=0.2276
