# Supplementary material for: Early 5‐HT 6 receptor blockade prevents symptom onset in a model of adolescent cannabis abuse
Source: EMBO Mol Med. 2020 Apr 24;12(5):e10605. doi: 10.15252/emmm.201910605 (PMC7207164; doi:10.15252/emmm.201910605)
Supplement: Supplementary file 2 — Expanded View Figures PDF [file EMMM-12-e10605-s002.pdf]

Expanded View Figures

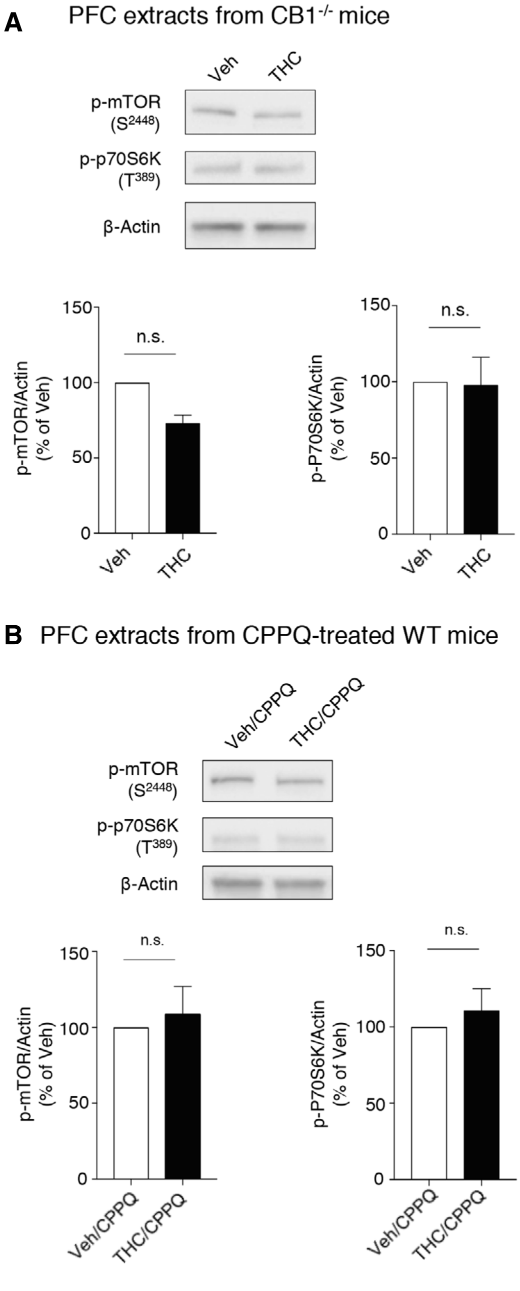

Figure EV1. Chronic intake of THC during adolescence does not induce mTOR activation in PFC of $CB_1^{-/-}$ mice and wild-type mice treated with CPPQ during adolescence.

A $CB_1^{-/-}$ mice were injected daily with THC (5 mg/kg) or vehicle (Veh) during adolescence, from PND 30 to 45. Top: representative Western blots assessing mTOR phosphorylation at S2448 and p70S6K phosphorylation at T389 as indexes of mTOR activity in the PFC of adult $CB_1^{-/-}$ mice are illustrated. Bottom: data represent the ratios of immunoreactive signals of the anti-phospho-mTOR (S2448) or anti-phospho-P70S6K (T389) antibodies to the immunoreactive signal of the anti-β-actin antibody and are expressed in % of values in vehicle-injected mice. They are the means ± SEM of results obtained in three mice per group. $P > 0.05$, unpaired Student's t test. n.s.: not significant.

B Wild-type mice were injected daily with THC (5 mg/kg) or vehicle (Veh) during adolescence, from PND 30 to 45. CPPQ (2.5 mg/kg) was administered concomitantly with vehicle or THC. Top: representative Western blots assessing mTOR phosphorylation at S2448 and p70S6K phosphorylation at T389 as indexes of mTOR activity in the PFC of adult WT mice are illustrated. Bottom: data represent the ratios of immunoreactive signals of the anti-phospho-mTOR (S2448) or anti-phospho-P70S6K (T389) antibodies to the immunoreactive signal of the anti-β-actin antibody and are expressed in % of values in vehicle-injected mice. They are the means ± SEM of results obtained in three mice per group. $P > 0.05$, unpaired Student's t test.

 © 2020 The Authors

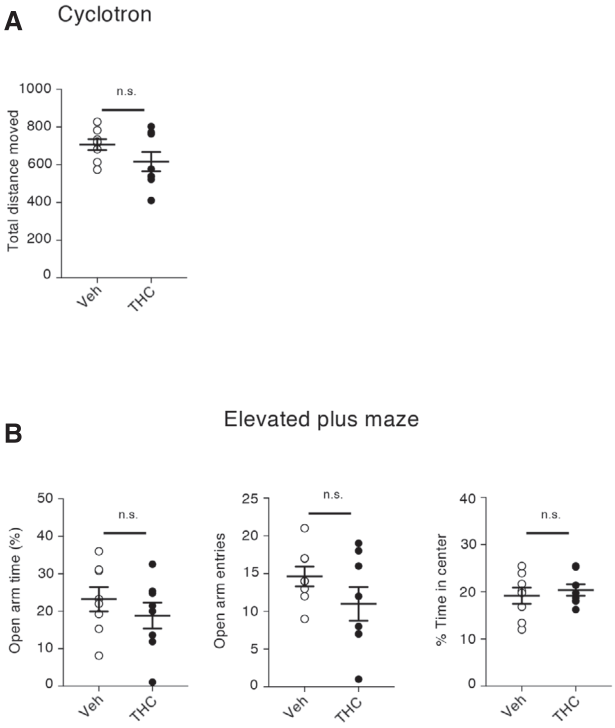

Figure EV2. Chronic THC intake during adolescence does not induce an alteration in locomotion nor anxiety-related behavior.

A Distance moved in the open field and percentage of time moving in the center of the open field. Distance moved: 706 ± 29 cm and 616 ± 51 cm for vehicle (N = 8) and THC (N = 8) conditions, respectively, $P > 0.05$, unpaired Student's t test. Time spent in the center: 19.18 ± 1.69% and 20.39 ± 1.22% for vehicle (N = 8) and THC (N = 8) conditions, respectively, $P > 0.05$, unpaired Student's t test. Errors bars correspond to the mean ± SEM.

B Percentage of open arm time and entries in the EPM. Time spent in the open arm: 23.24 ± 3.25% and 18.86 ± 3.45% for vehicle (N = 8) and THC (N = 8), respectively, $P > 0.05$, unpaired Student's t test. n.s.: not significant. Number of entries in the open arm: 15 ± 1 entries and 11 ± 2 entries for vehicle (N = 8) and THC (N = 8) conditions, respectively, $P > 0.05$, unpaired Student's t test. Errors bars correspond to the mean ± SEM.

© 2020 The Authors

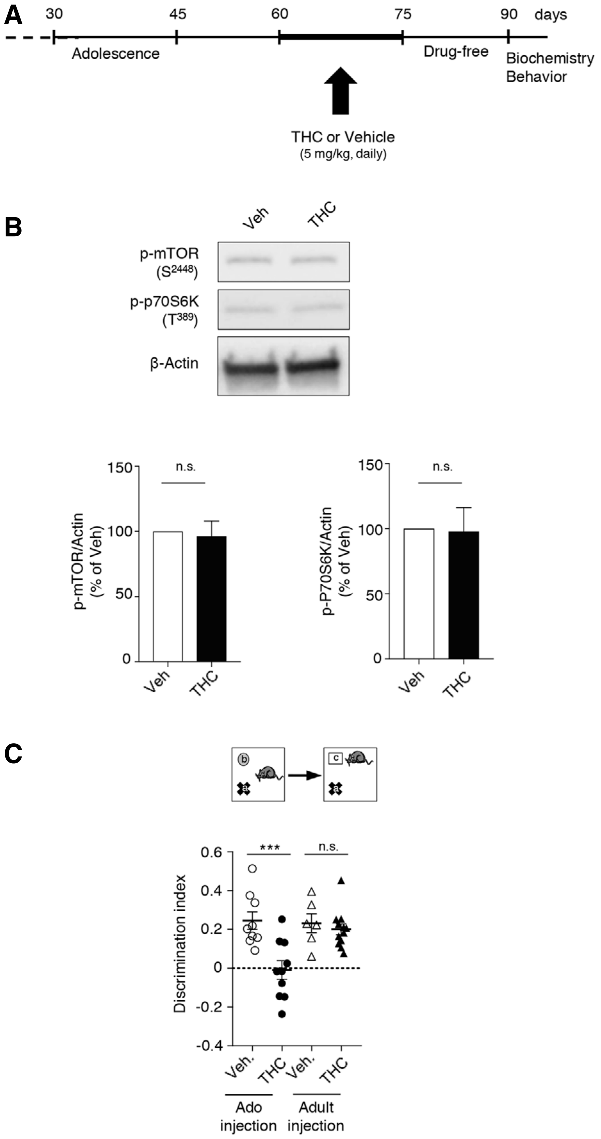

Figure EV3. Chronic THC administration to adult mice does not induce sustained mTOR activation and cognitive deficits.

A Schema of the experimental paradigm used for drug administration. Mice were injected daily with THC (5 mg/kg) or vehicle (Veh) in adulthood, from PND 60 to 75. Biochemical and behavioral experiments were performed from PND 90.

B Top: representative Western blots assessing mTOR activity in PFC are illustrated. Bottom: data represent the ratios of immunoreactive signals of the anti-phospho-mTOR (S2448) or anti-phospho-p70S6K (T389) antibodies to the immunoreactive signal of the anti-β-actin antibody and are expressed in % of values in vehicle-injected mice. They are the means ± SEM of results obtained in five mice per group. $P > 0.05$, one-way ANOVA followed by Newman–Keuls test. n.s.: not significant.

C The plots represent the discrimination index for the novel object recognition task measured in each condition (new groups of THC or vehicle-injected mice during adolescence were performed). The discrimination index for the novel object recognition task is 0.24 ± 0.05 ($N = 9$) and −0.01 ± 0.05 ($N = 10$), for mice treated with vehicle and THC during adolescence, respectively, and 0.23 ± 0.05 ($N = 6$) and 0.20 ± 0.03 ($N = 12$), for mice treated with vehicle and THC in adulthood, respectively. ***$P < 0.001$ and n.s. $P > 0.05$, one-way ANOVA followed by Bonferroni test. Error bars correspond to the mean ± SEM and the dotted line to a discrimination index equal to zero.

 © 2020 The Authors

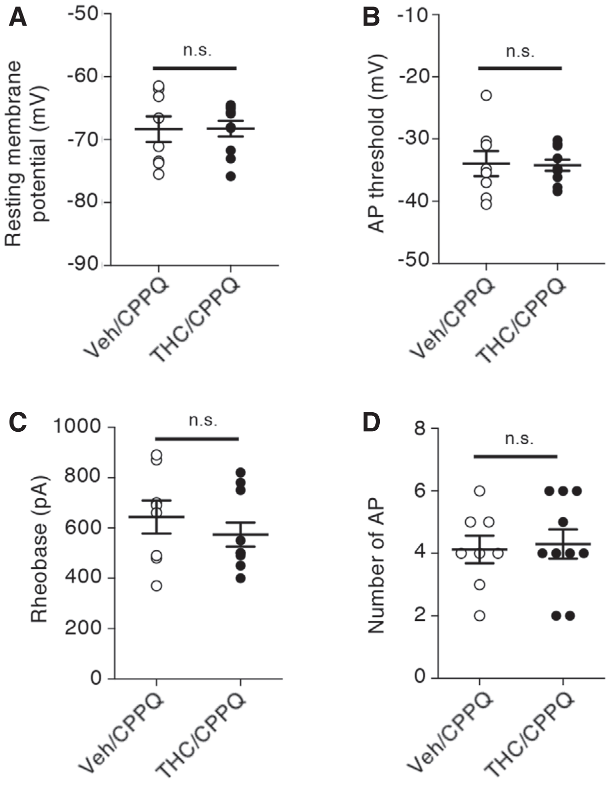

Figure EV4. Administration of CPPQ during adolescence prevents the alterations of layer V pyramidal neuron intrinsic properties.

A–D Mice injected daily with either vehicle (Veh) or THC from PND30 to 45 were concomitantly treated with CPPQ (2.5 mg/kg, Veh/CPPQ: $n = 8$ from $N = 4$ and THC/CPPQ: $n = 10$ from $N = 4$). Electrophysiological recordings were performed from PND 60. The plots represent means ± SEM of RMPs, AP thresholds, rheobases, and firing rates (measured as described in the legend to Fig 5), respectively. n.s.: $P > 0.05$, unpaired Student's t test. RMP: −68.4 ± 2.0 and −68.3 ± 1.2 mV for Veh/CPPQ and THC/CPPQ, respectively; AP threshold: −33.9 ± 2.0 and −34.2 ± 0.9 mV for Veh/CPPQ and THC/CPPQ conditions, respectively; Rheobase: 644 ± 66 and 574 ± 48 pA for Veh/CPPQ and THC/CPPQ conditions, respectively.

© 2020 The Authors
