# Supplementary material for: Early 5‐HT 6 receptor blockade prevents symptom onset in a model of adolescent cannabis abuse
Source: EMBO Mol Med. 2020 Apr 24;12(5):e10605. doi: 10.15252/emmm.201910605 (PMC7207164; doi:10.15252/emmm.201910605)
Supplement: Supplementary file 6 — Source Data for Figure 3 [file EMMM-12-e10605-s005.pdf]

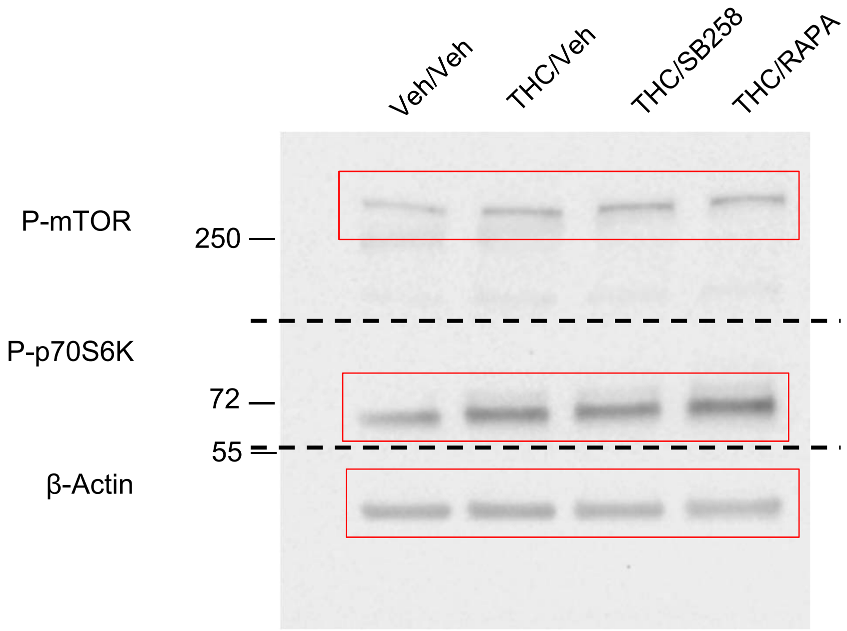

Western blot related to Figure 3

Note that the membrane was cut to blot it with different antibodies.
The cuts are indicated by the dtted lines.
